# Distinct Molecular Epidemiology, Transmission Patterns, and Resistance Mutations of HIV-1 Subtypes A1, A6, and A7 in Bulgaria

**DOI:** 10.3390/microorganisms13051108

**Published:** 2025-05-12

**Authors:** Aleksandra Partsuneva, Anna Gancheva, Reneta Dimitrova, Lyubomira Grigorova, Asya Kostadinova, Maria Nikolova, Radoslava Emilova, Nina Yancheva, Rusina Grozdeva, Ivailo Alexiev

**Affiliations:** 1 National Reference Confirmatory Laboratory of HIV, National Center of Infectious and Parasitic Diseases (NCIPD), 1504 Sofia, Bulgaria; alexandra.partsuneva@gmail.com (A.P.); gancheva.anna@gmail.com (A.G.); naydenova.reneta@gmail.com (R.D.); lyubomiragrigorova@gmail.com (L.G.); deshova.asi@gmail.com (A.K.); 2 National Reference Laboratory of Immunology, National Center of Infectious and Parasitic Diseases (NCIPD), 1504 Sofia, Bulgaria; mr_nklv@yahoo.com (M.N.); remilova@ncipd.org (R.E.); 3 Specialized Hospital for Active Treatment of Infectious and Parasitic Diseases, 1606 Sofia, Bulgaria; nyancheva@gmail.com (N.Y.); rusina.s.grozdeva@gmail.com (R.G.)

**Keywords:** HIV-1, subtype A1, subtype A6, subtype A7, molecular epidemiology, phylogenetic analysis, resistance mutations, Bulgaria

## Abstract

By 2022, Bulgaria’s National Reference Laboratory had confirmed 4024 HIV cases. We analyzed 132 pol gene sequences to characterize the molecular epidemiology of HIV-1 subtypes A1, A6, and A7 (2001–2022). A1 accounted for 50.0% (66/132) of cases, increasing after 2014, with peaks in 2019 and 2022. A6 comprised 48.5% (64/132), dominating from 2005 to 2014 before stabilizing. A7 was rare (1.5%, 2/132), detected in 2003 and 2011. Transmission patterns varied: A1 was linked to men who have sex with men (MSM) (62.1%), while A6 was primarily heterosexual (HET) (82.8%) with a balanced gender distribution (56.3% male, 43.8% female). Resistance mutations were identified in 29.6% of cases, with A6 showing higher rates of nucleoside reverse transcriptase inhibitor (NRTI) (20.3%) and non-nucleoside reverse transcriptase inhibitor (NNRTI) (7.8%) resistance than A1. Phylogenetic analysis revealed that 13 Bulgarian sequences (9.8%) were involved in transmission clusters, including 10 (7.6%) from sub-subtype A1 and 3 (2.3%) from sub-subtype A6, highlighting distinct genetic diversity and transmission patterns. Despite significant migration from Ukraine in 2022, A6 prevalence remained unchanged, suggesting localized transmission dynamics. These findings highlight a shifting HIV-1 sub-subtype distribution in Bulgaria and emphasize the need for targeted prevention, diagnosis, and treatment strategies tailored to the evolving molecular landscape.

## 1. Introduction

Human immunodeficiency virus type 1 (HIV-1) remains a critical global health concern, continuing to spread rapidly across populations worldwide. HIV-1 is classified into four distinct phylogenetic groups: M (main), N (new), O (outlier), and P, each originating from independent cross-species transmission events from nonhuman primates to humans [1]. Group M, responsible for the ongoing pandemic, is the predominant group and encompasses multiple genetically distinct subtypes (A–D, F–H, J, K and L). Some of these subtypes further diversify into sub-subtypes (e.g., A1–A4, A6–A8, F1, F2), circulating recombinant forms (CRFs), and unique recombinant forms (URFs) [2,3]. The unequal geographic distribution and distinct dissemination patterns of these subtypes, CRFs, and URFs are likely driven by diverse founder effects, followed by regional spread influenced by socioeconomic factors such as population growth, migration, and urbanization, as well as behavioral factors such as transmission within vulnerable groups [4,5]. These dynamics contribute to the extensive genetic diversity and variability of HIV-1, which complicates diagnosis, viral load measurements, and antiretroviral therapy (ART) efficacy, and may promote the development of HIV drug resistance (HIVDR), posing a significant challenge to HIV vaccine development [6].

Subtype A is prevalent across Eastern Europe, East Africa, and Central Asia, including Russia and the countries of the former Soviet Union (FSU). The sub-subtypes A1 and A6 are closely related, with A6 emerging as a descendant of A1, originating in Africa, primarily in Rwanda, Kenya, Uganda, and Tanzania. Sub-subtype A7 was identified in Nigeria through complete genome and pol gene analysis [7,8,9]. Subtype C predominates in Southern Africa, India, and Ethiopia, whereas subtype B is more prevalent in North and Latin America, Oceania, and Western and Central Europe. Globally, subtype C accounts for approximately 46% of infections, followed by subtype B (12%) and subtype A (10%) [2,10,11]. Central Africa exhibits the greatest diversity of CRFs and URFs due to the co-circulation of multiple subtypes, a trend also observed in Central Europe [2,12,13].

In Bulgaria, a significant diversity of HIV-1 subtypes, CRFs, and URFs has been documented, likely due to the country’s strategic geographic location at the intersection of Western and Eastern Europe, Africa, and the Middle East [14,15,16,17,18,19,20]. Most Balkan countries, including Bulgaria, underwent significant political and socioeconomic transformations following the end of the Cold War [14]. Additionally, the large-scale migration within FSU countries following the collapse of the Soviet Union in the early 1990s contributed to the rapid spread of HIV-1. During the epidemic in the Soviet Union, the newly classified sub-subtype A6 (previously referred to as AFSU) was repeatedly transmitted [8,21]. The recent conflict between Russia and Ukraine has significantly contributed to migration patterns associated with the transmission of HIV-1 subtype A6 [16]. It is believed that subtype A and its subdivisions were introduced into Bulgaria multiple times over the years. Understanding the origin and dissemination of HIV-1 subtype A and its sub-subtypes in Bulgaria has critical epidemiological and treatment relevance.

## 2. Materials and Methods

### 2.1. Study Design and Specimen Preparation

In this study, we analyzed 132 HIV-1 subtype A sequences obtained from patients diagnosed between 2001 and 2022 at the National Reference Confirmatory Laboratory of HIV (NRCL of HIV) of the National Center of Infectious and Parasitic Diseases (NCIPD) in Sofia, Bulgaria. At the time of diagnosis, demographic and epidemiological data were collected through a standardized survey in accordance with national regulations. To ensure confidentiality, the demographic and clinical data of each patient were anonymized using unique digital codes, in line with ethical standards in the Republic of Bulgaria. Blood samples were processed via centrifugation, and the plasma was stored at −80 °C in individual tubes, which were labeled with the sample receipt date, as described in previous study [15].

### 2.2. Sequence Analysis and Dataset

For this study, the HIV-1 pol gene was sequenced from 132 patient samples using the ViroSeq HIV-1 Genotyping Test (Abbott, Chicago, IL, USA) and/or the TruGene DNA Sequencing System (Siemens Healthcare, Erlangen, Germany). Sequencing was performed using an Applied Biosystems 3130xl Genetic Analyzer (Waltham, MA, USA) and/or the OpenGene DNA Sequencing System (Siemens), following the manufacturers’ protocols [15].

HIV-1 subtype A was identified in the sequences using the automated web-based tools COMET v2.4 and REGA v3.0. Manual phylogenetic analysis was conducted using reference sequences downloaded from the Los Alamos HIV Sequence Database (https://www.hiv.lanl.gov/content/index/, accessed on 4 May 2025), which confirmed the presence of subtypes A1, A6, and A7 [22,23]. For the subsequent phylogenetic analyses, sequences from various Balkan countries (Greece, Slovenia, Croatia, Romania, Turkey, Albania, Montenegro, and Serbia) and other regions globally (including Russia, Ukraine, Germany, Congo, Kenya, Nigeria, and Rwanda) were retrieved from the Los Alamos database and integrated.

An additional BLAST search was performed on the selected sequences isolated at the NRCL of HIV for subtypes A1, A6, and A7 to identify the closest phylogenetically related sequences, which were subsequently incorporated into the analysis. Sequence alignment of the Los Alamos reference sequences, BLAST results, and Bulgarian sequences was conducted using the MUSCLE algorithm within AliView version 1.17.1 [24,25].

The phylogenetic relationships of the final alignment were determined using the maximum likelihood (ML) method, implemented via the IQ-TREE v1.6.12 web server [26]. Tree reconstruction was performed using IQ-TREE v1.6.12. An initial tree was generated using a parsimony starting tree from the phylogenetic likelihood library. Model selection was carried out using IQ-TREE’s ModelFinder across 88 candidate nucleotide substitution models. The best-fit model, TVM + F + I + G4, was selected according to the Bayesian Information Criterion (BIC). Node support was evaluated using 1000 ultrafast bootstrap replicates [27,28]. The resulting tree was midpoint-rooted and used for subsequent cluster analyses. Tree visualization was performed with FigTree v1.4.4. This alignment included a total of 398 sequences: 132 from Bulgaria, 22 reference subtype sequences, and 244 specifically selected sequences, spanning 905 nucleotides.

Cluster analysis was performed using the ClusterPicker software to identify potential transmission clusters within the sub-subtype A clades [29]. Phylogenetic clusters were defined based on an intra-cluster genetic distance threshold of 1.5% (0.015 nucleotide substitutions per site), indicating both recent and more distant transmission events [30]. The initial cluster support threshold was set to a bootstrap value ≥ 90%.

HIV-1 resistance mutations conferring resistance to protease inhibitors (PIs) NRTI and NNRTI were determined with the HIVdb Program: Mutations Analysis Tool version 9.7, Stanford University HIV Drug Resistance Database and Sierra algorithm for inference of drug resistance (https://hivdb.stanford.edu/hivdb/by-patterns/ accessed on 4 May 2025).

### 2.3. Statistical Analysis

The statistical analysis was conducted to assess the differences in demographic, epidemiological, and clinical characteristics across HIV-1 sub-subtypes A1, A6, and A7. Categorical variables were analyzed using the Chi-square test when all expected frequencies in the contingency tables exceeded five. This approach was applied to variables such as gender distribution (male vs. female), likely routes of HIV transmission (heterosexual contact, men who have sex with men, people who inject drugs, and dual exposure), country of infection (domestic vs. abroad), and the presence of sexually transmitted infections (STIs). Resistance mutation frequencies were also analyzed using the Chi-square test when data permitted.

For categories with small sample sizes or sparse data, such as certain subcategories within routes of HIV transmission (e.g., dual exposure) and resistance mutations with zero or low frequencies, Fisher’s exact test was employed. This test was chosen for its robustness in handling low-frequency data and small contingency tables, particularly for sub-subtype A7, which had limited observations.

Statistical significance was determined at a threshold of *p* < 0.05. Highly significant results, with *p*-values less than 0.001, were reported as <0.001, while non-significant results were reported to three decimal places to ensure precision. The statistical analyses were performed using appropriate software, and the choice of test for each comparison was based on the distribution and size of the data.

## 3. Results

### 3.1. Study Population

A total of 132 HIV-1 subtype A sequences were analyzed, obtained from patients diagnosed between 2001 and 2022. Among these, 66 (50.0%) were classified as HIV-1 sub-subtype A1, 64 (48.5%) as sub-subtype A6, and 2 (1.5%) as sub-subtype A7 (Table 1). The median age of participants at diagnosis was 36.8 years. Of the cohort, 98 (74.2%) individuals were male and 34 (25.8%) were female. Gender distribution varied significantly by sub-subtype. Sub-subtype A1 was predominantly associated with male patients (90.9%), with a female representation of only 9.1%. In contrast, sub-subtype A6 exhibited a more balanced distribution, with 56.3% of participants being male and 43.8% female.

Four primary HIV transmission routes were identified based on self-reported data: HET accounted for 78 cases (59.1%), MSM for 50 cases (37.9%), people who inject drugs (PWID) for 3 cases (2.3%), and 1 individual (0.8%) reported dual exposure (MSM + PWID). Notably, MSM was the predominant transmission route among sub-subtype A1 cases (41, 62.1%), followed by HET (23, 34.8%) and PWID (2, 3.0%). Conversely, HET transmission was dominant among sub-subtype A6 cases (53, 82.8%), with fewer cases attributed to MSM (9, 14.1%) and one case (1.6%) involving PWID and MSM + PWID.

Foreign acquisition of infection was reported by 7 individuals (10.6%) with sub-subtype A1 and 27 individuals (42.2%) with sub-subtype A6. These cases included Bulgarian nationals infected abroad and non-national individuals diagnosed with HIV in Bulgaria. All individuals in this group were categorized as migrants based on self-reported data, which indicated that both the Bulgarian nationals and the non-national individuals reported acquiring their HIV infection outside of Bulgaria.

Coinfection with other sexually transmitted infections (STIs) was reported by 17 individuals (25.8%) with sub-subtype A1 and only 5 individuals (7.8%) with sub-subtype A6.

Of the 132 participants, 115 (87.1%) were ART-naive at the time of sequencing. Among ART-naive individuals, resistance mutations were detected in 34 cases (29.6%): 1 (0.9%) had protease inhibitor (PI) resistance, 19 (16.5%) had NRTI resistance, and 21 (18.3%) had NNRTI resistance. Dual-class resistance to both NRTIs and NNRTIs was observed in 7 samples. Among the 17 individuals with prior ART exposure, resistance mutations were identified in 7 cases (41.2%): 6 (35.3%) showed NRTI resistance, 4 (23.5%) had NNRTI resistance, and 2 (11.8%) exhibited resistance to both NRTIs and NNRTIs.

Phylogenetic analysis revealed 34 clusters (25.8%), with a higher prevalence in sub-subtype A1 (24 clusters, 36.4%) compared to significantly fewer clusters in sub-subtype A6 (8 clusters, 12.5%) (Table 1).

### 3.2. Statistical Analysis of the Study Characteristics

The statistical analysis revealed key variations in characteristics across HIV-1 sub-subtypes A1, A6, and A7, providing insights into the demographic, epidemiological, and clinical profiles of the study population.

A highly significant difference in gender distribution was observed across subtypes (*p*-value < 0.001). Sub-subtype A1 was predominantly associated with male participants (90.9%), while sub-subtype A6 exhibited a more balanced distribution (56.3% male, 43.8% female). This indicates a substantial gender disparity between the two sub-subtypes.

For the route of HIV transmission, statistical significance varied. The *p*-value for HET was 0.100, indicating no significant variation in HET proportions across subtypes. In contrast, other transmission routes, such as MSM and PWID, yielded more significant results, with *p*-values of <0.001 for both MSM and PWID. Small sample sizes, particularly for dual exposure (MSM + PWID), may have limited the power of these comparisons.

The analysis of sexually transmitted infection (STI) prevalence demonstrated a statistically significant association with HIV-1 subtypes (*p*-value = 0.009). Sub-subtype A1 had a higher frequency of STI co-infection compared to A6, suggesting subtype-specific patterns in co-infection risk.

For resistance mutations, statistical significance was observed for nucleoside reverse transcriptase inhibitors (NRTIs) and non-nucleoside reverse transcriptase inhibitors (NNRTIs), with *p*-values of <0.001 for both. These findings highlight differing resistance patterns between subtypes. However, small sample sizes in certain mutation categories may have affected the ability to detect statistically significant differences in some cases.

The phylogenetic analysis revealed a statistically significant difference in the number of clusters (*p*-value < 0.001), with sub-subtype A1 having more clusters than A6. This suggests that sub-subtype A1 is more genetically diverse in this cohort.

### 3.3. Phylogenetic Clusters

**Figure 1:**
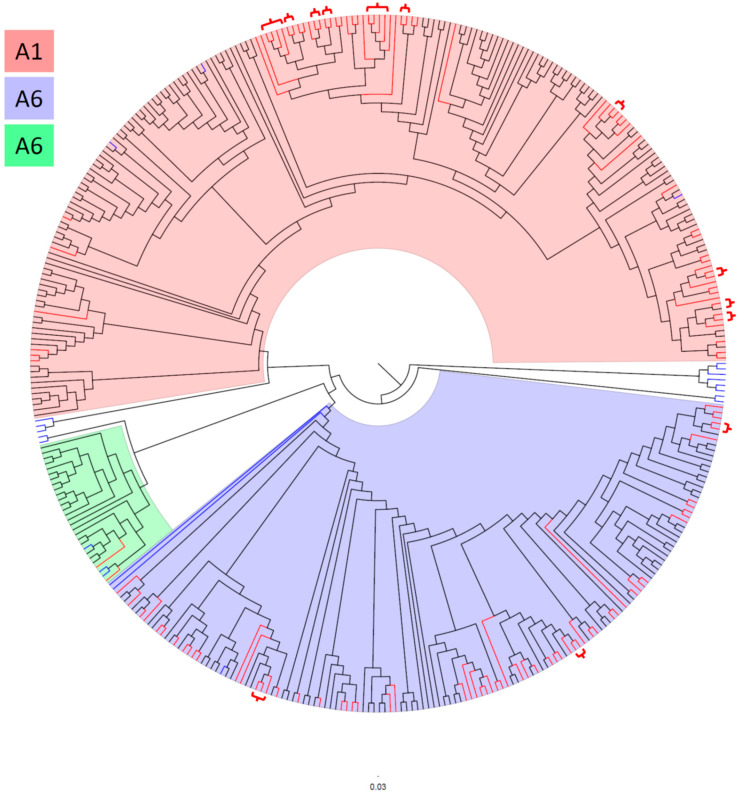
Maximum-likelihood (ML) phylogenetic tree of HIV-1 subtype A sequences. The ML tree was reconstructed using IQ-TREE v1.6.12 with a total of 398 sequences: 132 Bulgarian sequences, 22 reference subtype sequences, and 244 specifically selected sequences. The analysis covered 905 nucleotide positions. Background colors indicate the clustering of HIV-1 sub-subtypes: blue represents sub-subtype A6, green represents sub-subtype A7, and pink represents sub-subtype A1. Clades without background shading represent reference sequences outside of these three sub-subtypes. Bulgarian sequences are colored in red, reference sequences in blue, and sequences from BLAST and the specially selected set from the Los Alamos database are shown in black. Red brackets on the phylogenetic tree indicate phylogenetic clusters involving Bulgarian sequences, and the purple bracket indicates a cluster involving two sequences, one Bulgarian and one Croatian.

To investigate the phylogenetic relationships between Bulgarian HIV-1 sub-subtypes A1, A6, and A7, and sequences from other countries, a total of 397 HIV-1 pol sequences were analyzed. This analysis aimed to identify potential phylogenetic clusters within the subtype A sub-epidemic in Bulgaria (Figure 1 and Figure S1).

A total of 34 phylogenetic clusters were identified in the phylogenetic tree. Among these, 13 (38.2%) clusters contained 30 (22.7%) Bulgarian sequences, comprising 27 sequences of sub-subtype A1 and 7 sequences of sub-subtype A6, see brackets on the phylogenetic tree (Figure 1). The largest clusters were observed in sub-subtype A1, where each cluster contained four sequences, whereas the largest cluster for sub-subtype A6 contained three sequences. In sub-subtype A1, 10 clusters were identified: two clusters contained four sequences each, while the remaining eight contained two sequences each. Three sub-subtype A6 clusters were identified: one cluster contained three sequences, while the other two contained two sequences each.

Transmission mode analysis of the 23 sub-subtype A1 sequences within clusters revealed that 13 originated from MSM, 8 from HET individuals, and 2 from PWIDs. Among the seven clustered sub-subtype A6 sequences, five were linked to HET, one to MSM, and one to PWID transmission.

Regarding the origin of infection, only 4 (3%) of the 132 individuals participating in clusters reported acquiring their infections outside of Bulgaria. Specifically, three Bulgarian nationals were infected abroad, and one foreign national was diagnosed in Bulgaria. In contrast, 32 (31.4%) of the 102 individuals who did not participate in clusters reported that their infections were likely acquired outside the country. Notably, 19 individuals who reported probable infection acquisition in Ukraine, Russia, or Belarus carried HIV-1 sub-subtype A6, which is characteristic of these countries. Of these 19 individuals, only one MSM was found in a cluster of four male participants, three of whom reported MSM transmission and one reported HET transmission.

Additionally, one Bulgarian sequence isolated from a PWID was found in a cluster with a sequence from Croatia, exhibiting 100% bootstrap support in the bootscan analysis conducted with IQ-TREE and ClusterPicker.

### 3.4. Distribution of the Sub-Subtypes

**Figure 2:**
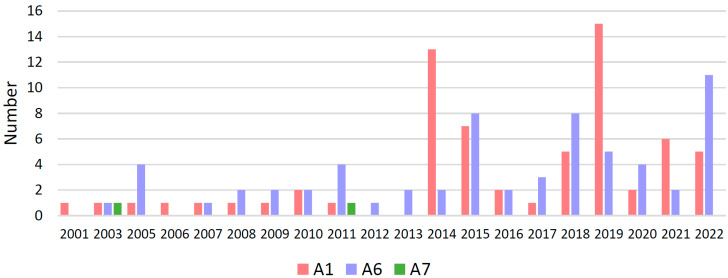
Temporal sub-subtype distribution. Number of individuals with HIV-1 subtype A1, A6, and A7 by year of diagnosis in Bulgaria.

The fluctuation in the distribution of HIV-1 sub-subtypes A1, A6, and A7 among individuals diagnosed in Bulgaria between 2001 and 2022 demonstrated distinct trends (Figure 2). Sub-subtype A1, represented in pink, exhibited a consistent upward fluctuation over the study period. Initially identified in low numbers during the early years, its frequency increased after 2014, with pronounced peaks in 2019 and 2022. This pattern suggests an increasing dominance of sub-subtype A1 in more recent years.

In contrast, sub-subtype A6, shown in blue in Figure 2, was relatively frequently encountered during the earlier years, particularly between 2005 and 2014. After 2015, its frequency stabilized, with notable occurrences in 2019 and 2022 but without evidence of a significant upward or downward trend. These findings indicate that both sub-subtypes A6 and A1 fluctuate and have uneven increases and decreases over the years.

Sub-subtype A7, represented in green, was detected only sporadically throughout the study period. It was identified in isolated cases in 2003 and 2011, but was otherwise absent in most years, showing no discernible trend in prevalence.

These results suggest a potential epidemiological shift in the distribution of sub-subtypes over time, characterized by the increasing predominance of A1. This shift may be attributed to changes in transmission dynamics, regional epidemiology, or other biological and behavioral factors influencing sub-subtype prevalence.

Despite the substantial migration of refugees from Ukraine following the onset of Russia’s war against Ukraine in 2022, there was no significant increase in the prevalence of sub-subtype A6 in our national surveillance data on the introduction and spread of HIV-1 subtypes in Bulgaria.

## 4. Discussion

This study presents a comprehensive molecular epidemiological analysis of HIV-1 subtypes A1, A6, and A7 in Bulgaria over a two-decade period, highlighting the temporal dynamics, transmission patterns, and resistance mutations associated with these sub-subtypes. The findings offer valuable insights into the evolving landscape of HIV-1 in Bulgaria, with implications for both public health surveillance and clinical management.

Our analysis revealed a distinct distribution of HIV-1 A sub-subtypes. Initially rare, A1 has shown a consistent upward trend since 2014, culminating in peaks in 2019 and 2022 [14]. This trend suggests an expanding transmission network for A1, potentially driven by changes in transmission dynamics or founder effects. Despite the significant presence of subtype A in some regions of the world, there is no trend for this subtype to increase globally [2]. Additionally, among subtype A1 cases, MSM was the predominant transmission route, and coinfection with other STIs was more common. In contrast, sub-subtype A6, which was the predominant clade during the early years of the study (2005–2014), has reached a plateau in prevalence. While A6 remains a prominent lineage, its relative contribution has diminished compared to A1, reflecting possible shifts in regional epidemiology or transmission patterns [8,21]. Sub-subtype A7, detected only sporadically, remains rare and shows no discernible trend, suggesting limited introductions and reduced transmission efficiency [31]. Despite the substantial influx of migrants from Ukraine following the onset of the Russia–Ukraine conflict in 2022, no significant increase in A6 prevalence was observed in Bulgaria until the end of 2022. [8,21,32]. This finding underscores the effectiveness of existing public health measures and suggests that local transmission dynamics remain the primary driver of HIV-1 sub-subtype distribution.

The demographic and transmission characteristics of sub-subtypes A1 and A6 were notably distinct. A1 was predominantly associated with MSM and male individuals, indicating a concentrated epidemic within this high-risk group. In contrast, A6 exhibited a more balanced gender distribution and was primarily associated with heterosexual transmission, highlighting its broader reach across various population segments [33]. The higher prevalence of STIs among A1 cases compared to A6 suggests differential co-infection risks, which may influence transmission dynamics [34,35]. These findings emphasize the importance of targeted interventions tailored to the unique profiles of each sub-subtype, particularly among vulnerable populations such as MSM.

The phylogenetic analysis identified 34 clusters, with sub-subtype A1 exhibiting significantly more clusters than A6. The greater clustering in A1 reflects its higher genetic diversity and more active transmission networks, likely driven by rapid transmission within MSM communities. Conversely, the fewer clusters in A6 indicate a more stable transmission pattern, consistent with its plateau in prevalence [36,37]. Interestingly, transnational clustering was rare, with only one instance of a Bulgarian sequence clustering with a Croatian sequence (HIV-1 isolate CRO1239 from Croatia, GenBank accession number MN163423). This suggests that local transmission accounts for the majority of clusters, reinforcing the importance of national surveillance efforts.

The study revealed significant differences in drug resistance patterns between sub-subtypes. Compared to A1, A6 exhibited higher rates of resistance mutations, particularly to NRTIs. This highlights the need for continued monitoring of resistance trends to ensure the effectiveness of ART. Although dual-class resistance is infrequent, it underscores the complexity of managing HIV-1 in ART-experienced individuals. In addition, some observations suggest that HIV-1 subtype A6/A1, in combination with other contributing factors, may be associated with an increased risk of virologic failure during long-acting cabotegravir and rilpivirine therapy [38,39,40]. This should be taken into consideration, and the prevalence of A6/A1 in Bulgaria, as well as the response to cabotegravir and rilpivirine therapy in these patients, should be closely monitored. The high proportion of ART-naïve individuals with resistance mutations further emphasizes the importance of resistance testing before initiating therapy [30,33].

The findings of this study have several public health implications. The increasing dominance of A1 necessitates focused interventions to address its concentrated transmission within MSM populations. Enhanced STI prevention and treatment efforts may also mitigate the co-infection risks associated with A1. For A6, maintaining robust surveillance is critical to monitor potential changes in its epidemiology, particularly in the context of regional migration. The observed resistance patterns highlight the need for tailored treatment strategies and underscore the importance of integrating resistance testing into routine clinical practice. Additionally, the low prevalence of transnational clusters suggests that localized interventions will remain effective in controlling the spread of HIV-1 in Bulgaria.

While this study provides critical insights, several limitations should be noted. First, the sample size for sub-subtype A7 was small, limiting the ability to draw robust conclusions about its epidemiology. Second, self-reported data on transmission routes and country of infection may be subject to recall bias or social desirability bias. Lastly, while the study included a diverse set of sequences, it is possible that some clusters or subtypes were underrepresented due to sampling limitations.

Maintaining careful molecular epidemiological surveillance of both cross-border and internal transmission, with particular attention to vulnerable groups and migrants, is essential for obtaining a timely and accurate picture of the development of the HIV epidemic in the country.

## 5. Conclusions

This study highlights the dynamic nature of the HIV-1 subtype A epidemic in Bulgaria, marked by the rising dominance of sub-subtype A1, the stabilization of A6, and the sporadic occurrence of A7. The distinct epidemiological profiles, transmission patterns, and resistance mutations associated with these sub-subtypes underscore the need for subtype-specific strategies in prevention, diagnosis, and treatment. Future research should explore the biological and behavioral factors driving these trends and assess the potential impact of emerging subtypes and recombinant forms. Expanding genomic surveillance and integrating advanced phylogenetic tools will be essential to track the evolution of HIV-1 and inform public health strategies aimed at achieving sustained epidemic control.

## Figures and Tables

**Table 1:** Patient characteristics in the study.

Patient Characteristic	Individuals in the Study with Subtype A	Sub-Subtype A1	Sub-Subtype A6	Sub-Subtype A7	*p*-Value
Total (n, %) *	132 (100)	66 (50.0)	64 (48.5)	2 (1.5)	-
**Gender**					<0.001
Male	98 (74.2)	60 (90.9)	36 (56.3)	2 (100)	
Female	34 (25.8)	6 (9.1)	28 (43.8)	0 (0.0)	
**Likely Route of HIV Infection**					0.100
HET	78 (59.1)	23 (34.8)	53 (82.8)	2 (100)	
MSM	50 (37.9)	41 (62.1)	9 (14.1)	0 (0.0)	<0.001
PWID	23 (34.8)	2 (3.0)	1 (1.6)	0 (0.0)	<0.001
MSM + PWID	1 (0.8)	0 (0.0)	1 (1.6)	0 (0.0)	<0.001
**Likely Country of Infection**					<0.001
Bulgaria	96 (72.7)	59 (89.4)	37 (57.8)	0 (0.0)	
Abroad	36 (27.3)	7 (10.6)	27 (42.2)	2 (100)	
**STI**					0.009
Yes	22 (16.7)	17 (25.8)	5 (7.8)	0 (0.0)	
No	110 (83.3)	49 (74.2)	59 (92.2)	2 (100)	
**Resistance Mutations**					<0.001
PR	1 (0.8)	0 (0.0)	1 (1.6)	0 (0.0)	
NRTI	16 (12.1)	3 (4.5)	13 (20.3)	0 (0.0)	<0.001
NNRTI	16 (12.1)	11 (16.7)	5 (7.8)	0 (0.0)	<0.001
NRTI + NNRTI	9 (6.8)	6 (9.1)	3 (4.7)	0 (0.0)	<0.001
**Total Sequences Participating in Clusters**	34 (25.8)	24 (36.4)	8 (12.5)	2 (100)	<0.001
**Bulgarian Sequences Participating in Cluster**	13 (9.8)	10 (7.6)	3 (2.3)	0	<0.001

* Percentages are calculated based on the total number of subtype A (A1, A6, A7) (132). *p*-value, *p* < 0.05: Indicates statistically significant differences.

## Data Availability

All HIV pol sequences generated in our study have been deposited in GenBank with the following accession numbers: EF517458, EF517475, EF517483, JQ259077, JQ259087, JQ259105, JQ259108, JQ259124, JQ259126, JQ259146, JQ259150, KJ765399, KJ765402, KJ765426, KJ765435, KJ765440, KJ765450, KJ765455, KJ765465, KJ765502, KJ765519, KJ765520, KJ765526, KJ765547, KJ765570, KJ765591, and PV105779-PV105884. Additional data presented in this study are available upon request from the corresponding author.

## Supplementary Materials

The following supporting information can be downloaded at: https://www.mdpi.com/article/10.3390/microorganisms13051108/s1, Figure S1. Maximum-likelihood (ML) phylogenetic tree of HIV-1 subtype A sequences.
