# Supplementary material for: Distinct Molecular Epidemiology, Transmission Patterns, and Resistance Mutations of HIV-1 Subtypes A1, A6, and A7 in Bulgaria
Source: Microorganisms. 2025 May 12;13(5):1108. doi: 10.3390/microorganisms13051108 (PMC12114506; doi:10.3390/microorganisms13051108)
Supplement: Supplementary file 1 [file microorganisms-13-01108-s001.zip › microorganisms-3584975-supplementary.pdf]

Supporting Materials

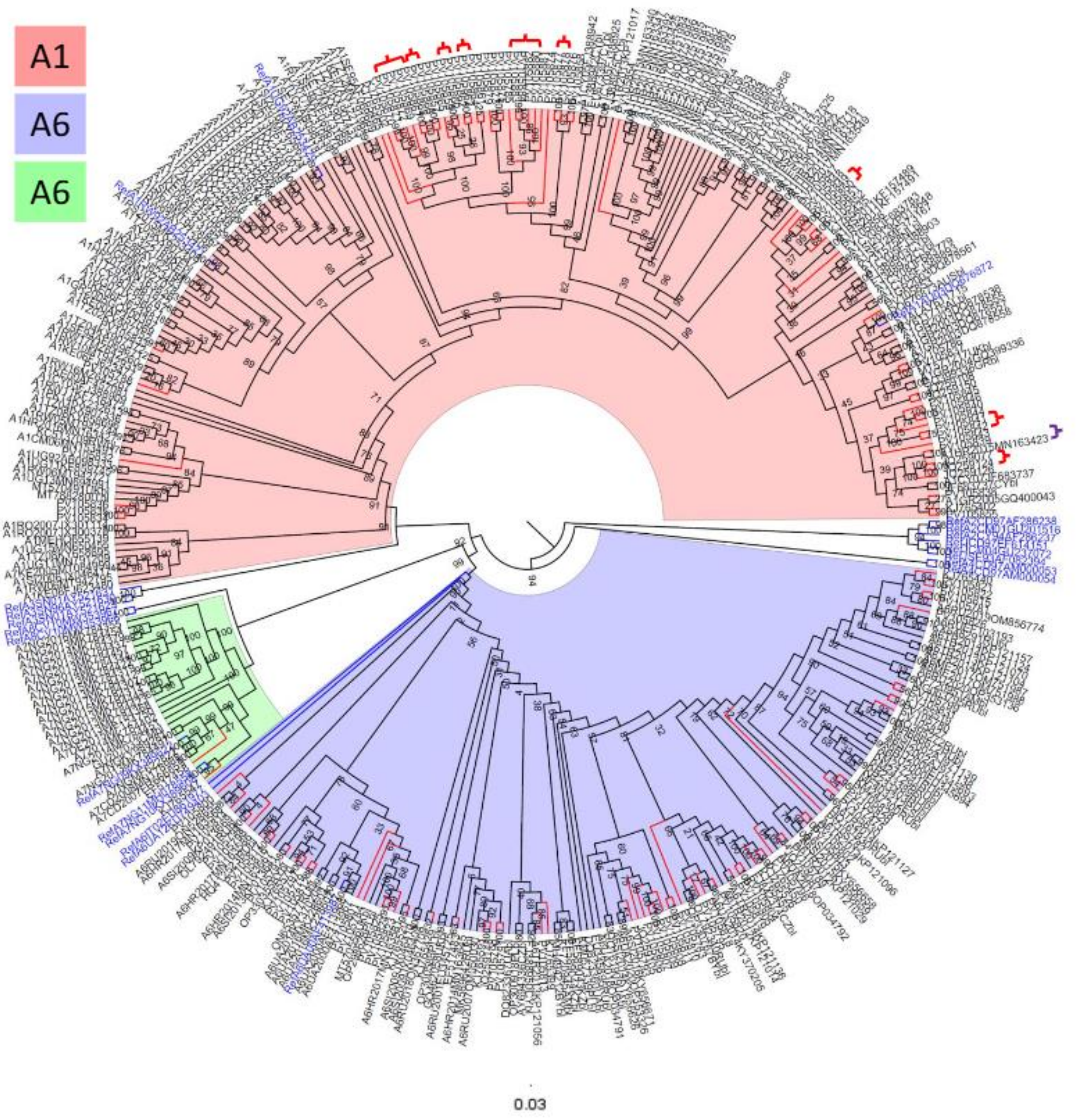

Figure S1. Maximum-likelihood (ML) phylogenetic tree of HIV-1 subtype A sequences. The tree was reconstructed using IQ-TREE v1.6.12 based on 905 nucleotide positions from a total of 398 sequences, including 132 Bulgarian sequences, 22 reference subtype sequences, and 244 specifically selected sequences. Bootstrap support values are shown at all nodes to indicate the statistical confidence of branching. Cluster affiliations of HIV-1 sub-subtypes are indicated by background shading: blue for sub-subtype A6, green for A7, and pink for A1. Clades that are not shaded represent reference sequences outside of the A1, A6, and A7 sub-subtypes. Sequence categories are color-coded as follows: Bulgarian sequences in red, reference sequences in blue, and sequences from BLAST and the Los Alamos database in black.
